# Supplementary material for: In Vitro and In Vivo Neuroprotective Effects of Stellettin B Through Anti-Apoptosis and the Nrf2/HO-1 Pathway
Source: Mar Drugs. 2019 May 29;17(6):315. doi: 10.3390/md17060315 (PMC6627894; doi:10.3390/md17060315)
Supplement: Supplementary file 1 [file marinedrugs-17-00315-s001.pdf]

Supplementary Materials:

In Vitro and In Vivo Neuroprotective Effects of Stellettin B Through Anti-Apoptosis and the Nrf2/HO-1 Pathway

Chien-Wei Feng [1,2,†], Nan-Fu Chen [3,4,†], Zhi-Hong Wen [2,5,†], Wen-Ya Yang [2], Hsiao-Mei Kuo [2,6], Ping-Jyun Sung [1,2,7], Jui-Hsin Su [1,7], Shu-Yu Cheng [5,8,9] and Wu-Fu Chen [2,9,10,*]

1 National Museum of Marine Biology and Aquarium, Pingtung 944, Taiwan; qscjuejuejue@gmail.com (C.-W. F.); pjsung@nmmba.gov.tw (P.-J. S.); x2219@nmmba.gov.tw (J.-H. S.)
2 Department of Marine Biotechnology and Resources, National Sun Yat-sen University, Kaohsiung 804, Taiwan; wzh@mail.nsysu.edu.tw (Z.-H. W.); s8222889@gmail.com (W.-Y. Y.); hsiaomeikuo@gmail.com (H.-M. K.)
3 Division of Neurosurgery, Department of Surgery, Kaohsiung Armed Forces General Hospital, Kaohsiung 802, Taiwan; chen06688@gmail.com (N.-F. C.)
4 Department of Neurological Surgery, Tri-Service General Hospital, National Defense Medical Center, Taipei 114, Taiwan
5 Doctoral Degree Program in Marine Biotechnology, National Sun Yat-sen University, Kaohsiung 804, Taiwan; joygetit@gmail.com (S.-Y. C.)
6 Center for Neuroscience, National Sun Yat-Sen University, Kaohsiung 804, Taiwan
7 Graduate Institute of Marine Biology, National Dong Hwa University, Pingtung 944, Taiwan
8 Doctoral Degree Program in Marine Biotechnology, Academia Sinica, Nankang, Taipei 115, Taiwan
9 Department of Neurosurgery, Kaohsiung Chang Gung Memorial Hospital and Chang Gung University College of Medicine, Kaohsiung 833, Taiwan
* Correspondence: ma4949@cgmh.org.tw; Tel.: 886-7-731-7123 (8011)
† These authors contributed equally to this work.

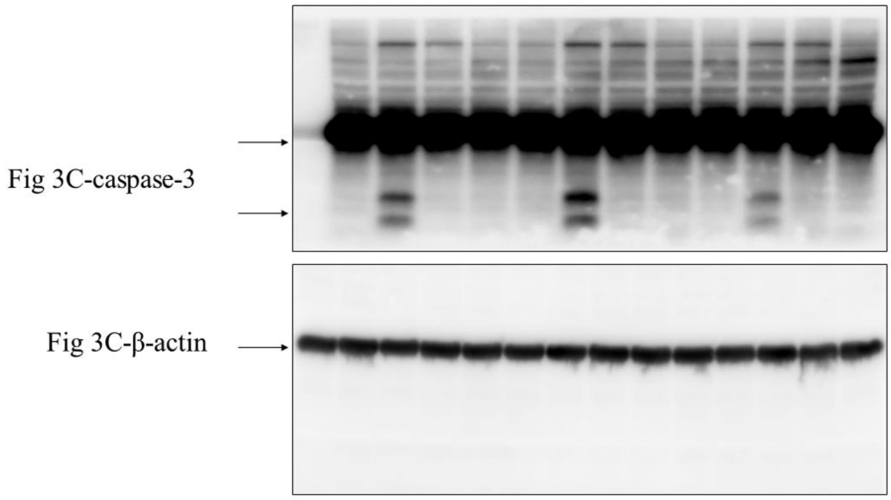

Figure S1: Uncropped Western blots of Fig 3C: caspase-3 and β-actin

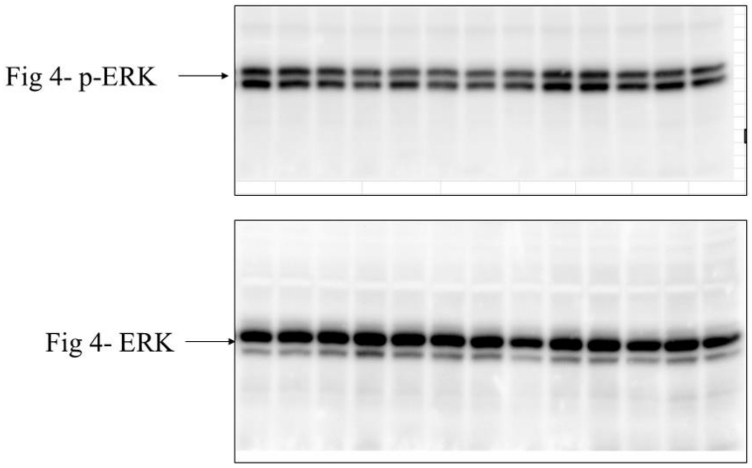

Figure S2: Uncropped Western blots of Fig 4: p-ERK and ERK

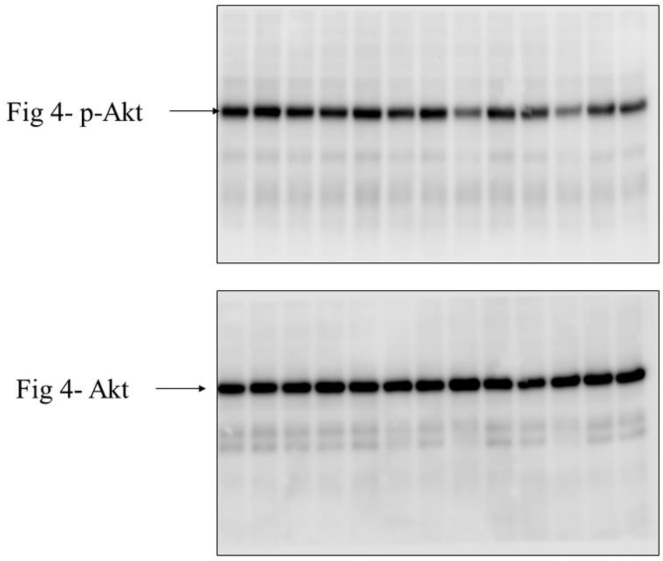

Figure S3: Uncropped Western blots of Fig 4: p-Akt and Akt

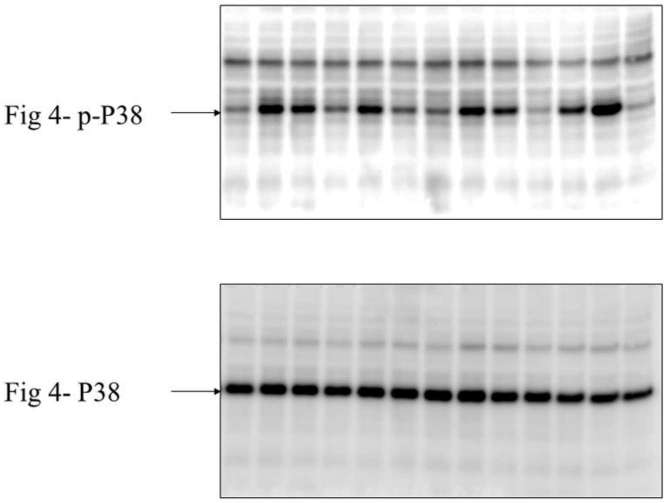

Figure S4: Uncropped Western blots of Fig 4: p-P38 and P38

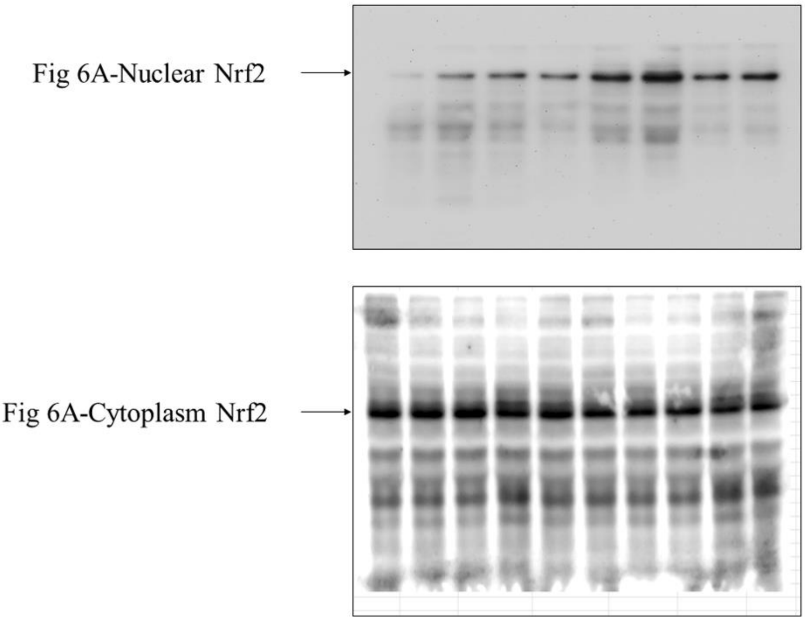

Figure S5: Uncropped Western blots of Fig 6A: Nuclear-Nrf2 and Cytoplasm-Nrf2

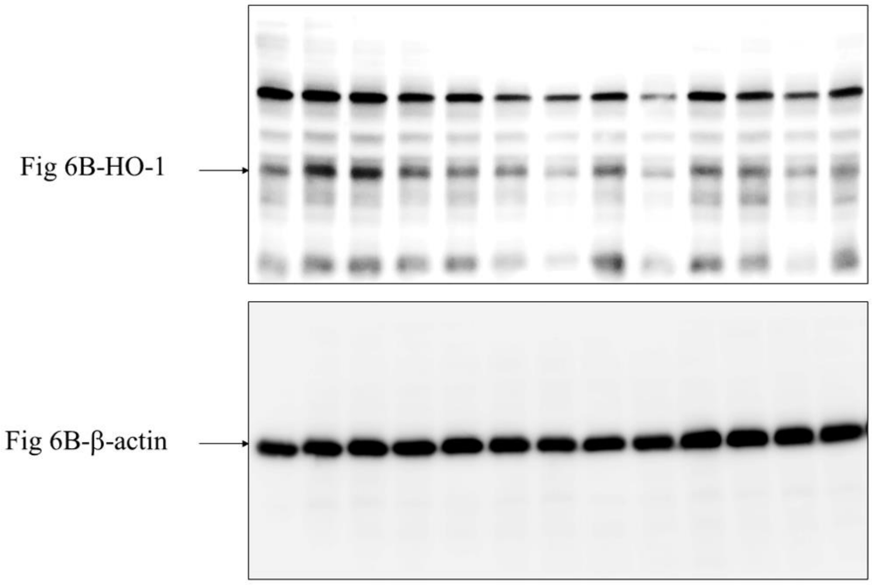

Figure S6: Uncropped Western blots of Fig 6B: HO-1 and β-actin

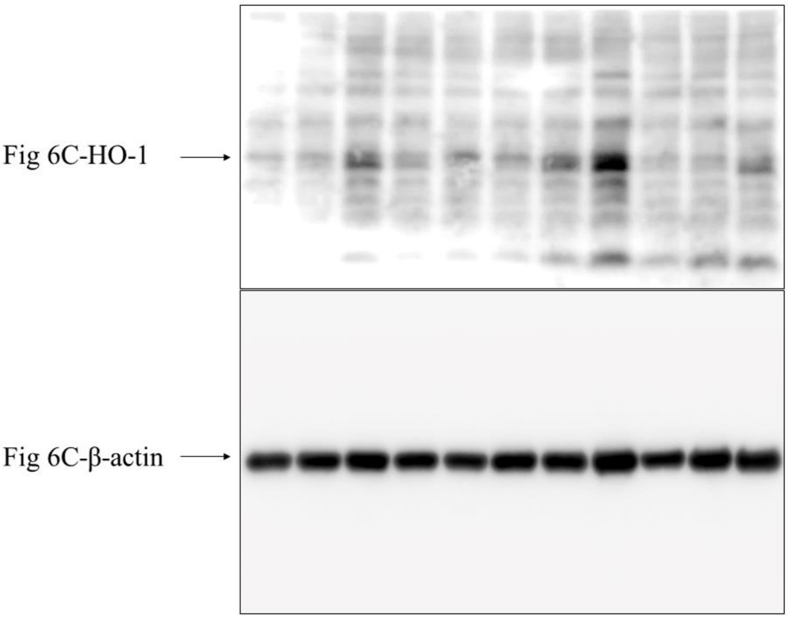

Figure S7: Uncropped Western blots of Fig 6C: HO-1 and β-actin

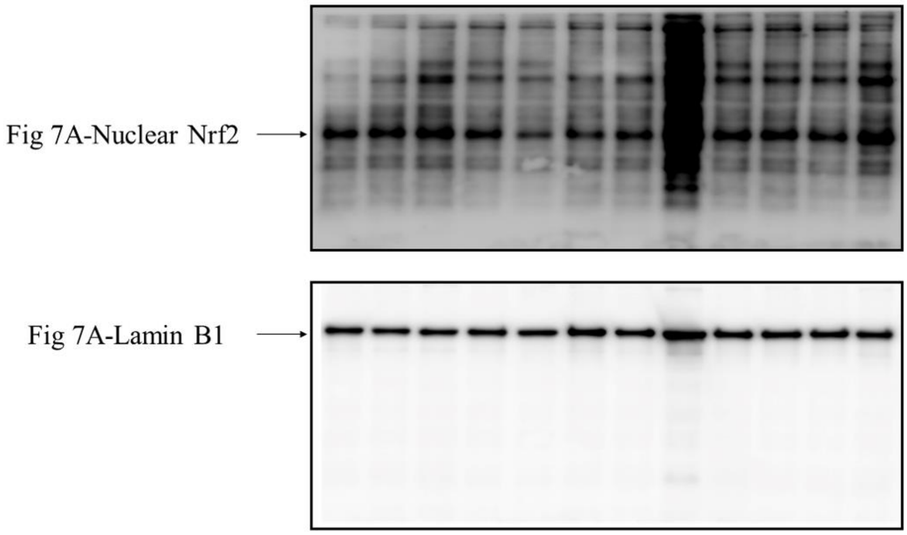

Figure S8: Uncropped Western blots of Fig 7A: Nuclear-Nrf2 and Lamin b1

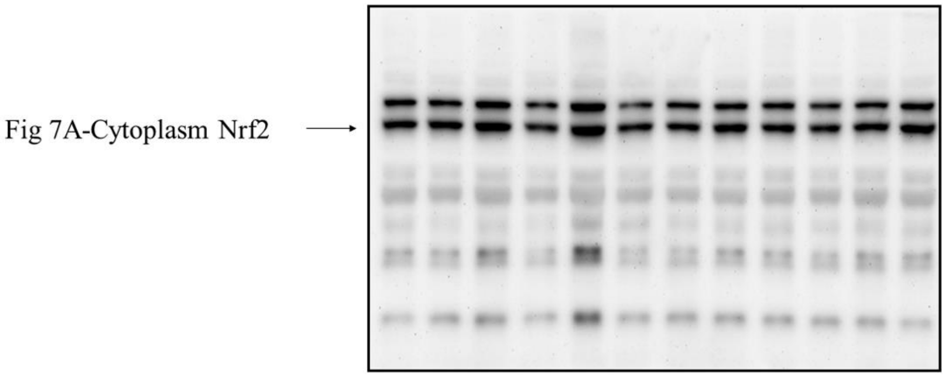

Figure S9: Uncropped Western blots of Fig 7A: Cytoplasm-Nrf2

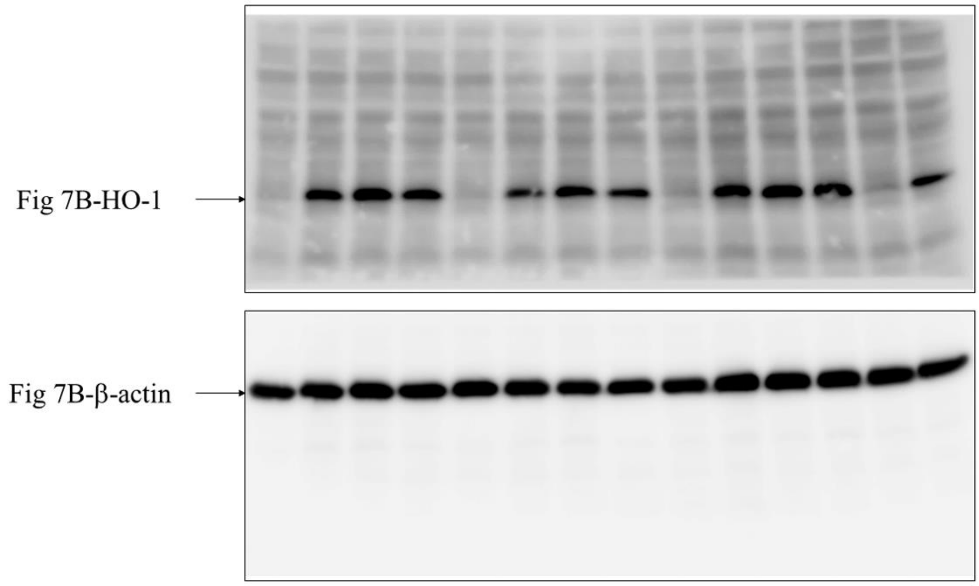

Figure S10: Uncropped Western blots of Fig 7B: HO-1 and β-actin

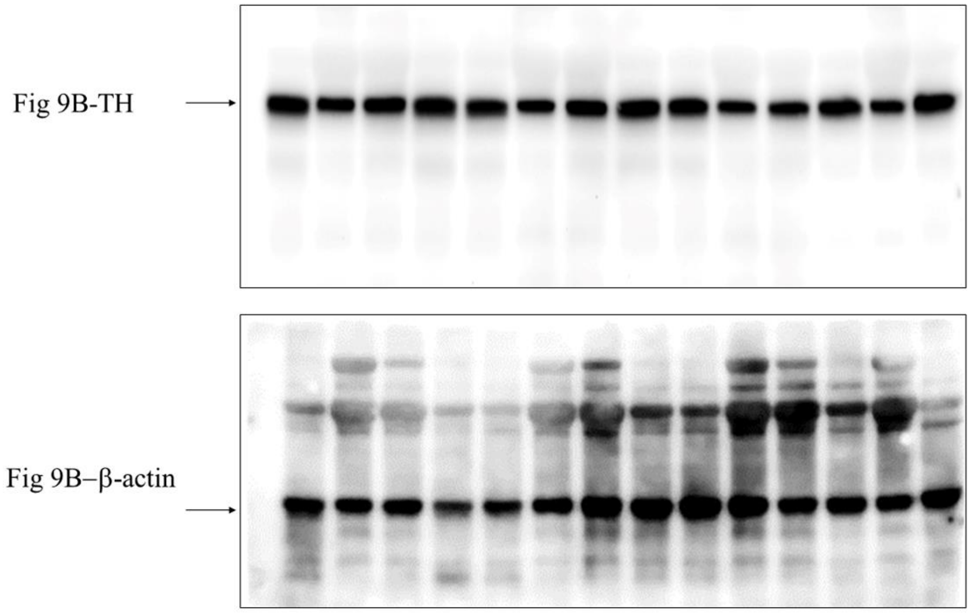

Figure S11: Uncropped Western blots of Fig 9B: TH and β-actin
